# Supplementary material for: Downsizing of COVID-19 contact tracing in highly immune populations
Source: PLoS One. 2022 Jun 10;17(6):e0268586. doi: 10.1371/journal.pone.0268586 (PMC9187098; doi:10.1371/journal.pone.0268586)
Supplement: S1 File — A, model description [16, 42, 43], [Table 1]. B, Contact tracing efficiency and controllable outbreaks. (PDF) [file pone.0268586.s001.pdf]

A Model description

A.1 The underlying SEAIR model

If c is the daily number of contacts per person, and α is the probability of a positive transmission given a contact, it follows that the number of people infected each day is given by:

$$\alpha c \, I \frac{S}{N} \, , \tag{3}$$

where variables S, I and N indicate the susceptible, infectious and total population. We differentiate infectious cases I into three subclasses, analogously to as done by Miller *et al* [16]: asymptomatic I_a (i.e., infectious individuals who will never display COVID-19 symptoms), pre-symptomatic I_p (i.e., infectious individuals who have not yet developed symptoms), and symptomatic cases I_c. The probability of an infection given a contact with an asymptomatic individual is lower than for symptomatic cases [42], and we assume that symptomatic individuals have a reduced contact rate, as individuals feeling sick are less likely to interact with others [16]. In the model, individuals in I_a, I_p and I_c have a different infectivity, quantified by parameters b_a, b_p and b_c respectively. Taking these factors into account, the number of people infected each day can be rewritten as:

$$\alpha c \, [b_p I_p + b_c I_c + b_a I_a] \frac{S}{N}, \tag{4}$$

where $b_a < b_p$ and $b_c < b_p$.

Infected individuals enter the exposed class E, and after a latency period of average length $1/\delta_E$ move either to the pre-symptomatic class I_p, with probability r, or to the asymptomatic class I_a, with probability $(1-r)$. Once symptoms occur, pre-symptomatic individuals enter the symptomatic class. After the disease runs its course, individuals enter the recovered class R.

A.2 Modelling Contact Tracing

We assume that only symptomatic individuals can contact trace, as these individuals are most likely to get tested once symptoms occur. We define the contact tracing efficiency q as the proportion of symptomatic individuals whose contacts will be traced, multiplied by the proportion of contacts of symptomatic individuals that are identified and quarantined. As only symptomatic individuals are contact traced, we write:

$$\begin{cases} q = 0 & \text{for} \quad I_c = 0 \, , \\ q = q_0 & \text{for} \quad I_c > 0 \, , \end{cases} \tag{5}$$

such that contract tracing is only activated in the presence of symptomatic infections (i.e., $I_c > 0$).

To identify contacts, we consider the interactions that individuals in I_c have had in the previous days with the susceptible population. Each symptomatic individual has entered class I_c by passing through the exposed class E and having entered the pre-symptomatic class with probability r. If we consider that the pre-symptomatic status lasts on average 2-3 days [16], and if we assume that symptomatic individuals become aware of their infectious

status 1-2 days after symptoms onset, then $r\delta_E E_{(t-5)}$ is number of people that entered I_p five days ago, and

$$I_c^{CT} = q[r\delta_E E_{(t-5)}] \tag{6}$$

is equivalent to the number of people whose contacts are being traced today.

Individuals whose contacts are being traced are sent to quarantine, as well as susceptible, exposed, asymptomatic and pre-symptomatic individuals that have interacted with them in the past five days. The total number of identified contacts that contact traced individuals have had with susceptible people in the past five days can be expressed as

$$S^{CT} = \underbrace{c}_{\substack{\text{contact}\\ \text{rate}}} \times \underbrace{q[r\delta_E E_{(t-5)}]}_{\substack{\text{individuals whose contacts}\\ \text{are being traced}}} \times \underbrace{\sum_{\tau=0}^{4} b_\tau \frac{S_{(t-\tau)}}{N}}_{\substack{\text{contacts}\\ \text{of the past 5 days}}} \tag{7}$$

where $b_\tau = b_p, b_c$ depending on whether the contact has been between susceptible and pre-symptomatic (meaning that at the time of the interaction the contact traced individual was still in I_p, i.e., $b_\tau = b_p$ for $\tau = 2, 3, 4$) or between susceptible and symptomatic cases (at the time of the interaction the contact traced individual had already entered I_c, i.e., $b_\tau = b_c$ for $\tau = 0, 1$).

As disease transmission given a contact occurs with probability α, a total of

$$\underbrace{(1-\alpha)c}_{\substack{\text{not-infected}\\ \text{contacts}}} \; q[r\delta_E E_{(t-5)}] \sum_{\tau=0}^{4} b_\tau \frac{S_{(t-\tau)}}{N} \tag{8}$$

people per day will have interacted with contact tracing individuals without developing an infection. These people will move from the susceptible class S to class S_q, which includes non-infectious individuals in isolation, and they will return to the susceptible class at the end of the quarantine period.

It follows that a total of

$$\alpha c \; q[r\delta_E E_{(t-5)}] \sum_{\tau=0}^{3} \; b_\tau \frac{S_{(t-\tau)}}{N} \tag{9}$$

individuals will develop the infection and enter the exposed class. Individuals who have interacted with contact traced individuals five days before contact tracing began, will have already left the exposed class, and therefore a total of

$$\alpha c \; q[r\delta_E E_{(t-5)}] \; b_p \frac{S_{(t-4)}}{N} \tag{10}$$

will be moved to quarantine after having already entered the pre-symptomatic class (with probability r) or the asymptomatic class (with probability $(1-r)$). All contact traced individuals developing an infection move to the class Q, and then enter the recovered class R at the end of the quarantine period.

Model equations: The system of delay differential equations representing the model dynamics can be written as:

$$\frac{dS}{dt} = \underbrace{-\alpha c\,[b_pI_p + b_cI_c + b_aI_a]\,\frac{S}{N}}_{S\to E} - \underbrace{(1-\alpha)cq[r\delta_E E_{(t-5)}]\sum_{\tau=0}^{4} b_\tau \frac{S_{(t-\tau)}}{N}}_{S\to S_q} + \underbrace{\delta_{S_q} S_q}_{S_q\to S}\,, \tag{11a}$$

$$\frac{dE}{dt} = \underbrace{\alpha c\,[b_pI_p + b_cI_c + b_aI_a]\frac{S}{N}}_{S\to E} - \underbrace{\alpha c\, q[r\delta_E E_{(t-5)}]\sum_{\tau=0}^{3} b_\tau \frac{S_{(t-\tau)}}{N}}_{E\to Q} - \underbrace{\delta_E E}_{E\to I_a, I_p}\;, \tag{11b}$$

$$\frac{dI_p}{dt} = \underbrace{r\delta_E E}_{E\to I_p} - \underbrace{r\alpha c\, q[r\delta_E E_{(t-5)}]\, b_p \frac{S_{(t-4)}}{N}}_{I_p\to Q} - \underbrace{\delta_{I_p} I_p}_{I_p\to I_c}\,, \tag{11c}$$

$$\frac{dI_c}{dt} = \underbrace{\delta_{I_p} I_p}_{I_p\to I_c} - \underbrace{q[r\delta_E E_{(t-5)}]}_{I_c\to Q} - \underbrace{\delta_{I_c} I_c}_{I_c\to R}\,, \tag{11d}$$

$$\frac{dI_a}{dt} = \underbrace{(1-r)\delta_E E}_{E\to I_a} - \underbrace{(1-r)\alpha c\; q[r\delta_E E_{(t-5)}] b_p \frac{S_{(t-4)}}{N}}_{I_a\to Q} - \underbrace{\delta_{I_a} I_a}_{I_a\to R}\,, \tag{11e}$$

$$\frac{dQ}{dt} = \underbrace{\alpha c\; q[r\delta_E E_{(t-5)}]\sum_{\tau=0}^{4} b_\tau \frac{S_{(t-\tau)}}{N}}_{E, I_a, I_p\to Q} + \underbrace{q[r\delta_E E_{(t-5)}]}_{I_c\to Q} - \underbrace{\delta_Q Q}_{Q\to R}\,, \tag{11f}$$

$$\frac{dS_q}{dt} = \underbrace{(1-\alpha)cq[r\delta_E E_{(t-5)}]\sum_{\tau=0}^{4} b_\tau \frac{S_{(t-\tau)}}{N}}_{S\to S_q} - \underbrace{\delta_{S_q} S_q}_{S_q\to S}\,, \tag{11g}$$

$$\frac{dR}{dt} = \underbrace{\delta_{I_c} I_c}_{I_c\to R} + \underbrace{\delta_{I_a} I_a}_{I_a\to R} + \underbrace{\delta_Q Q}_{Q\to R}\;. \tag{11h}$$

Note that the total number of individuals N remains constant over time. The flow diagram representation of Eq. (11) is presented in Fig 1. Default parameters are given in Table 1. We will consider the case where the community is initially virus-free, and thus at time zero $S = S_0$ (Eq. (12)), $E = 1$, and all other variables will be set to zero. Simulations will be performed with the dde23 solver of the software MATLAB R2018a. Because the system of Eq. (11) presents a discontinuity in parameter q (see Eq. (5)), for the simulations we choose to substitute the Heaviside step function with the continuous inverse tangent function shown in Fig A1. The computer code is publicly available at
https://figshare.com/articles/figure/Code_for_Figures_of_the_manuscript/19103183.

Contact tracing capacity: We assume that there is a limit in the contact tracing capacity, reached when the daily number of contacts to be traced (see Eq. (7) for $q = 1$) is higher than a certain value determined by I_{CT}^{max}. We will investigate the circumstances under which contact tracing is overwhelmed, and establish a criteria to determine the minimal contact tracing efficiency needed to avoid a major outbreak. We understand a controllable outbreak

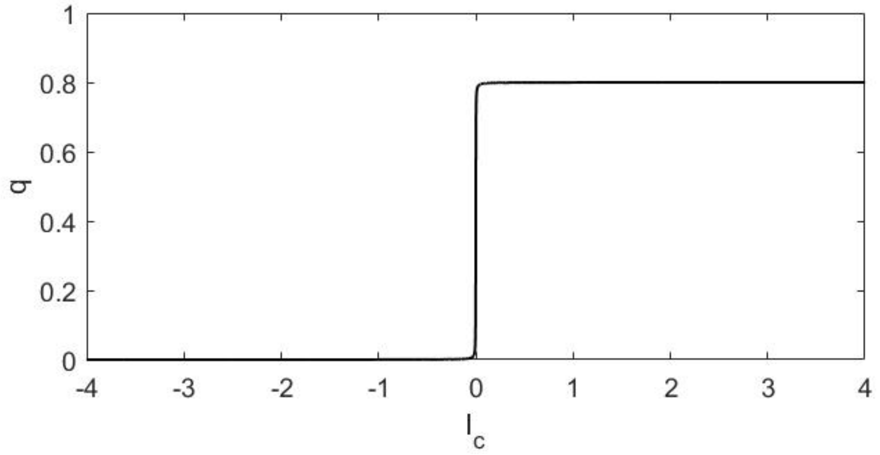

Figure A1: Inverse tangent function used for the numerical simulations to address the discontinuity in parameter q shown in Eq. (5). The function used is: $q(I_c) = q_0[1/\pi \arctan(1000(I_c - I_c^*)) + \pi/2]$, where here $q_0 = 0.8$ and $I_c^* = 0$.

as an outbreak where the daily number of contacts to be traced per day remains below the contact tracing capacity I_{CT}^{max} for T days. We will evaluate the minimal contact tracing efficiency required to keep an outbreak under control, with respect to the proportion of immune individuals and with respect to the contact rate c. Default values of I_{CT}^{max} and T are provided in Table 1.

A.3 Modelling immunity

To simulate differences in the immunity status of the population we assume that immunity reduces the initial size of the susceptible population S_0, where:

$$S_0 = (1 - p_v)N + p_v N(1 - \epsilon_v)\,. \tag{12}$$

The parameter p_v represents the proportion of the susceptible population that is immune (i.e., either vaccinated or recovered from infection). We assume that immunity reduces disease transmission with probability ϵ_v, and we consider $\epsilon_v = 0.89$ [43]. We assume that the immunity status of the population does not vary during the simulation period.

A.4 Modelling delays in outbreak detection

Often outbreaks in a community are not promptly detected, and the disease can spread uncontrolled for several days before measures are introduced. We analyse this scenario by considering that contact tracing is activated only when a certain number of symptomatic cases I_c^* is found in the community. We modify the contact tracing efficiency q, defined in Eq. (5), as follows:

$$\begin{cases} q = 0 & \text{for} \quad I_c \leq I_c^*, \\ q = q_0 & \text{for} \quad I_c > I_c^*\,. \end{cases} \tag{13}$$

We will compute the daily number of contacts to be traced for different I_c^*, as a function of the proportion of immune individuals in a population and of the contact rate, to understand how vaccine roll-out and social distancing impact the contact tracing capacity needed to keep an outbreak under control.

A.5 Modelling case importations

To evaluate the health risks following the relaxation of border restrictions during vaccine roll-out, we investigate how case importations affect the daily number of contacts to be traced, for different proportions of immune individuals in a population and for different contact rates. When only one import is observed, we consider only one exposed case at the beginning of the simulation. For multiple imports m, we consider the m exposed cases as distributed evenly over T (where the first imports occurs at $t = 0$, the second import occurs at $t = T/m$, the third at $t = 2T/m$, and so on).

A.6 Quarantine effectiveness

By comparing the cumulative number of infectious and non-infectious people in quarantine, we can gain information about quarantine effectiveness, intended as the percentage of individuals quarantined that develop an infection. We will investigate how quarantine effectiveness varies with the proportion of immune individuals in a population, and with the probability of infection given a contact (α), which can differ, for example, when considering different variants of the SARS-CoV2 virus.

We calculate the quarantine effectiveness as the number of people in quarantine that develop an infection Q_I divided by the total number of quarantined people Q_{TOT}. The number of quarantined individuals that develop an infection corresponds to the cumulative number of individuals entering class Q (see. Eq. (11f)), i.e.:

$$Q_I = \int_0^T \left\{ \left[\alpha c\, q[r\delta_E E_{(t-5)}] \sum_{\tau=0}^{4} b_\tau \frac{S_{(t-\tau)}}{N} \right] + q[r\delta_E E_{(t-5)}] \right\} \mathrm{d}t. \tag{14}$$

To account for contacts between immune and contact traced individuals we compute the cumulative size of S_q (i.e., the number of people S_q^C in quarantine that will never develop the infection) as follows:

$$S_q^C = \int_0^T \left\{ \left[(1-\alpha) c q[r\delta_E E_{(t-5)}] \sum_{\tau=0}^{4} b_\tau \frac{S_{(t-\tau)}}{N} \right] \left(1 + \frac{N - S_0}{S} \right) \right\} \mathrm{d}t\,, \tag{15}$$

where the fraction $(N - S_0)/S$ accounts for immune contacts in quarantine.

Hence, we obtain that the quarantine effectiveness Q_{eff} can be computed as:

$$Q_{eff} = \frac{Q_I}{Q_I + S_q^C}\,. \tag{16}$$

Quarantine effectiveness depends therefore on the probability of infection given a contact (α) and on the proportion of immune individuals in a population, expressed through the number of susceptible individuals S_0. Note that quarantine effectiveness does not depend on the contact tracing efficiency q, as when individuals are quarantined the contact tracing efficiency q corresponds to the constant q_0 (see Eq. (5) for $I_c > 0$), and can therefore be taken out of the integrals of Eqs. (14) and (15) and simplified in Eq. (16).

B Contact tracing efficiency and controllable outbreaks

B.1 Minimal contact tracing efficiency

The minimal contact tracing efficiency q_0^* required to avoid a growth in the number of cases can be approximated analytically. By assuming that the number of infectious cases remains small over time, we can consider a substantially simplified version of the model of Eq. (11) consisting of a single differential equation in I, where I includes pre-symptomatic, symptomatic and asymptomatic infectious cases present in the time-dependent proportions p_{I_p}, p_{I_c} and p_{I_a} respectively. We write:

$$\frac{dI}{dt} = \underbrace{\alpha c \tilde{b} I \frac{S_0}{N}}_{\text{infected}} - \underbrace{\tilde{\delta} I}_{\text{recovered}} - \underbrace{\left(q\alpha c [r\delta_E E_{(t-5)}] \sum_{\tau=0}^{4} b_\tau \frac{S(t-\tau)}{N} + q[r\delta_E E_{(t-5)}] \right)}_{\text{quarantined}}, \tag{17}$$

where S_0 is the size of the susceptible population, which depends on immunity status (see Eq. (12)), parameter $\tilde{b}$ is defined as the weighted average of the contact rate (i.e., $\tilde{b} = p_{I_p} b_p + p_{I_c} b_c + p_{I_a} b_a$), and parameter $\tilde{\delta}$ is the average time spent in the infectious state, and can be computed as:

$$\tilde{\delta} = r \left(\frac{1}{\delta_{I_p}^{-1} + \delta_{I_c}^{-1}} \right) + (1-r)\delta_{I_a} \, . \tag{18}$$

The factor $q[r\delta_E E_{(t-5)}]$ is the number of individuals contact tracing today, which, for I small, can be approximated as:

$$q[r\delta_E E_{(t-5)}] \simeq q I_c = q p_{I_c} I \, . \tag{19}$$

We obtain that $dI/dt \leq 0$ as long as:

$$q \geq \frac{\alpha c \tilde{b} \frac{S_0}{N} - \tilde{\delta}}{p_{I_c} \alpha c (2b_c + 3b_p) \frac{S_0}{N} + p_{I_c}} \, . \tag{20}$$

Where the minimal contact tracing efficiency q_0^* required to avoid epidemic spread corresponds to the value of q for which equality in Eq. (20) is obtained. Eq. (20) indicates that q_0^* depends on the proportion of immune individuals in a population, given by the ratio S_0/N; the contact rate c; the probability of infection given a contact α; the contact rates of pre-symptomatic, symptomatic and asymptomatic individuals b_p, b_c and b_a; the average length of the infectious status $\tilde{\delta}$; and the proportion of symptomatic individuals p_{I_c}. A graphical representation of Eq. (20) is provided in Fig B1.

B.2 Contact tracing capacity

Simulations show that increasing contact tracing capacity only minimally affects the minimal contact tracing efficiency needed to avoid a major outbreak (i.e., the value of q_c^*, Fig B2).

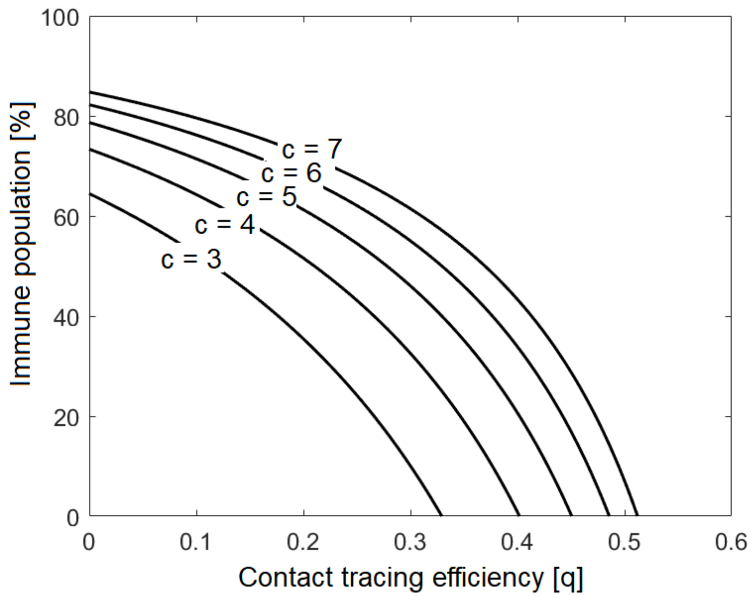

Figure B1: Analytical approximation of the minimal contact tracing efficiency q_0^* needed to avoid disease spread, as a function of the on the proportion of immune individuals and for different contact rates. The value of q_0^* is computed by considering equality in Eq. (20), for the contact rates $c = \{3, 4, 5, 6, 7\}$. Default parameters are given in Table 1.

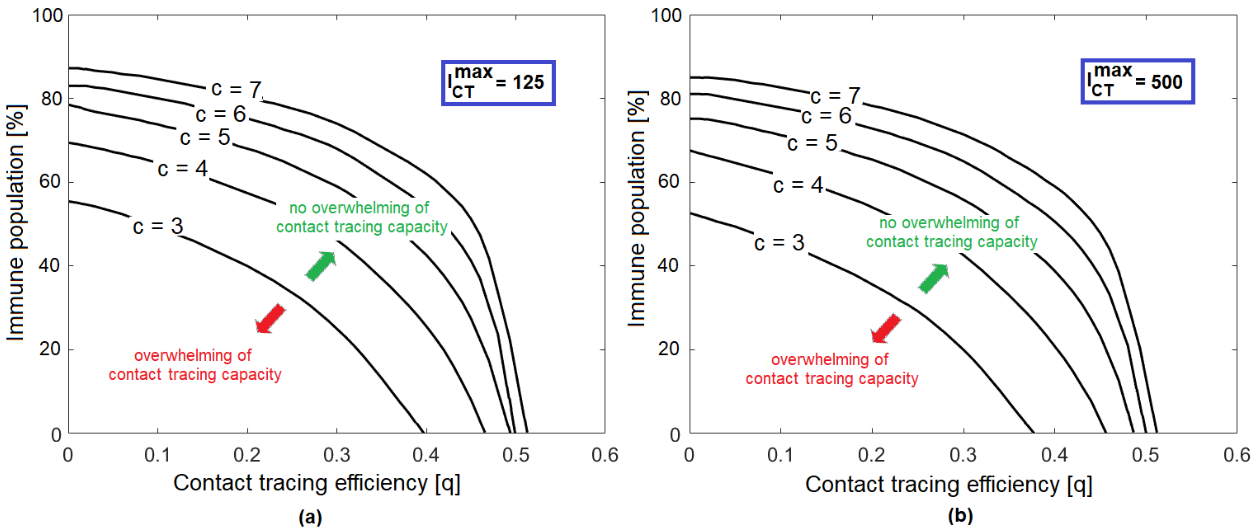

Figure B2: Contact tracing efficiency needed to avoid overwhelming contact tracing capacity, as a function of the proportion of immune individuals and for different contact rates (black curves, with $c = \{3, 4, 5, 6, 7\}$) for (a) a higher contact tracing capacity ($I_{CT}^{max} = 500$) and (b) a lower contact tracing capacity ($I_{CT}^{max} = 125$). The area below each curve represents the parameter space for which contact tracing is overwhelmed, while the area above each curve represents the parameter space for which contact tracing is not overwhelmed. The curves represent the minimal contact tracing efficiency q_c^* needed to avoid overwhelming contact tracing capacity. Parameter $I_c^* = 0$ for all simulations, other default parameters are given in Table 1. We see that, as explained in section 3.1, differences in the contact tracing capacity I_{CT}^{max} do not significantly affect the results (cfr. Eq. (20)).
