# Supplementary material for: Downsizing of COVID-19 contact tracing in highly immune populations
Source: PLoS One. 2022 Jun 10;17(6):e0268586. doi: 10.1371/journal.pone.0268586 (PMC9187098; doi:10.1371/journal.pone.0268586)
Supplement: Attachment — Submitted filename: PONE-D-21-34590_review.pdf [file pone.0268586.s002.pdf]

Downsizing of contact tracing during COVID-19 vaccine roll-out

23rd December 2021

1 Summary

The paper "Downsizing of contact tracing during COVID-19 vaccine roll-out" investigates the interplay between vaccination and contact tracing in controlling the still ongoing COVID-19 pandemic. The authors model the epidemic dynamics with a system of delay-differential equations which extends a previously published model by quarantine orders due to contact tracing and the effects of vaccination. Within this model the authors, by solving the system of delayed differential equations numerically, explore in several scenarios what measures are necessary to avoid overwhelming the capacities of contact tracing (and thus a major outbreak). The main messages of the paper are then that for contact tracing to be effective it has to be quick and efficient, vaccination supports contact tracing by reducing the efficiency of tracing necessary to keep the epidemic under control and that imported cases in populations with low vaccination quotas may quickly overwhelm contact tracing capacities. The paper is well written and its methods and derivations are easy to understand and follow. The findings of the paper are largely supported by the evidence that the authors present. I recommend that the paper is accepted after the authors address the following points.

2 Major Remarks

1. The title of the paper and the discussion suggest that the downsizing of contact tracing occurs simultaneously to the roll-out in of the vaccine and lifting of contact restrictions. Contrasting this, the paper deals with the situation where a fixed amount of the population is already vaccinated, contacts remain constant and contact tracing is only implemented after the fact. This should be formulated more explicitly in the title as well as introduction and discussion section of the paper.

2. The conclusion after Equation (17), namely that q^* is always smaller than 1 is mistaken. Indeed, take p_c very small, then the right-hand-side of

Equation (17) blows up. Additionally for q^* to be known analytically one would have to calculate the time-varying probabilities $p_{I_c}, p_{I_p}, p_{I_a}$ which, to my knowledge, one cannot. The authors should thus formulate this more carefully in the discussion section of the paper, especially the sentence "Analogously, we show that, when highly efficient, contact tracing alone can be considered an effective control measure even in the absence of vaccination or social distancing".

3. While some of the parameters in Table 1 stem from references, others are based on estimates or choice of the authors, e.g. the total population size N, the probability of infection given a contact α, the contact rate c and the amount of imported cases m. Indeed, a back-of-the-envelope calculation (setting q to 0) yields

$$R_0 \approx c\, r \left(\frac{b_p}{\delta_{I_p}} + \frac{b_c}{\delta_{I_c}} \right) + c\,(1-r) \frac{b_a}{\delta_{I_a}} = 5.5$$

which seems more in line with the Delta variant than the original strain. The text should elaborate more on the reasons for these specific values, ideally citing studies (especially for the critical parameters α and c).

4. The contact tracing efficiency q is never formally introduced and differs in meaning across the paper; in Equation (5) it is meant as the fraction of symptomatic cases whose contacts are traced while in Section 4 it is interpreted as the fraction of contacts of infected cases that are traced. While this distinction does not matter mathematically it should either be clarified what the interpretation of q is or argued that one can interpret it both ways.

5. If Equation (11) is to be the "cumulative size of S_q", then it is missing an integral (from 0 to T). Indeed setting $S_0 = N$ we see that Equation (11) is the same as the first term in Equation (9g). The same reasoning applies to the Section on quarantine effectiveness in the Appendix (e.g. the right-hand sides of Equations (18) and (19) still depend on t). The authors should check how this affects the simplification presented in Equation (19) (one cannot cancel E_{t-5} anymore) and the results presented in Figure 4.

3 Minor Remarks

1. In Section 3.1 the parameter p_{I_c} is sometimes called p_c, this should be unified.

2. Before Equation (17) one actually requires $\alpha\ c\ \frac{S_0}{N}$ to be big (relative to $\tilde{\delta}$ and p_c) to arrive at Equation (17); so higher vaccination coverage and lower probability of infection make the approximation worse.

3. The right-hand side of the system of equations (9a-9h) is discontinuous, as q depends on I_c and, when considered, imported cases cause discontinuities in the solution. Clarification on how the authors dealt with these discontinuities would allow the presented results to be more reproducible.

4. The reference Miller et al. 2020 has already been published (albeit under a slightly different name), the authors should cite the published version.

5. Some references, e.g. Davies et al. 2020, Davis et al 2021, Ferretti et al 2020, use "covid-19" and "sars-cov-2" instead of "COVID-19" and "SARS-CoV-2", the authors should fix the spelling in the references.

6. The beginning of Section 2.2 reads "As only symptomatic individuals are contact traced ...". With the more widespread availability of rapidtests pre- and asymptomatic cases can be identified early on which would allow to have their contacts traced as well. I suggest that the authors add a rationale on why only symptomatic individuals are contact traced.
