# Supplementary material for: Downsizing of COVID-19 contact tracing in highly immune populations
Source: PLoS One. 2022 Jun 10;17(6):e0268586. doi: 10.1371/journal.pone.0268586 (PMC9187098; doi:10.1371/journal.pone.0268586)
Supplement: Attachment — Submitted filename: Response to reviewers.pdf [file pone.0268586.s003.pdf]

Dear Editor,

We want to thank the reviewers for a thorough review of the manuscript titled: ‘Downsizing of contact tracing during COVID-19 vaccine roll-out’.

We have addressed the reviewers’ comments and believe that the paper has significantly improved as a result. As suggested by reviewer #2, we have changed the title of the manuscript to 'Downsizing of COVID-19 contact tracing in highly immune populations'.

Specific comments are answered below. Reviewers’ comments are reported in black, while our responses are in blue. The line numbers in our responses refer to the revised manuscript. Changes from the original manuscript have been highlighted in yellow in the pdf file.

Reviewers' (1) comments:

1. The model established in this work seems novel to me, at least I have not encountered a similar model for contact tracing yet. However, the main idea, namely considering transmission rates as a product of the average number of contacts is similar as done by Lipsitch et al. in [1], later followed e.g. by [2,3,4]. Up to my knowledge, it was the work by Lipsitch et al. where quarantine was considered this way in a compartmental model. I suggest the authors cite this paper or maybe also some of the ones using a similar method. An important difference between the present work and that of Lipsitch et al. is that in this paper, instead of contact with infected individuals, people are quarantined based on contact with contact traced people who are among those recently developing symptoms, i.e. among those who contracted the disease 5 days earlier. In the model, this is introduced correctly and (up to my knowledge) in a novel way. The authors then study the effect of various factors related the epidemic. However, the findings are not validated by application to real world situations.

[1] Lipsitch, M., Cohen, T., Cooper, B., Robins, J. M., Ma, S., James, L., et al. (2003). Transmission dynamics and control of severe acute respiratory syndrome. Science, 300(5627), 1966e1970.

[2] Safi, M. A., & Gumel, A. B. (2010). Global asymptotic dynamics of a model for quarantine and isolation. Discrete and Continuous Dynamical Systems - Series B, 14(1), 209e231.

[3] Mubayi, A., Kribs Zaleta, C., Martcheva, M., & Castillo-Chavez, C. (2010). A cost-based comparison of quarantine strategies for new emerging diseases. Mathematical Biosciences and Engineering, 7(3), 687e717.

[4] Barua, S., Dénes, A., Ibrahim, M. A., A seasonal model to assess intervention strategies for preventing periodic recurrence of Lassa fever, Heliyon 7(8):e07760(2021).

We thank the reviewer for mentioning these interesting papers. Indeed, these are very relevant to our work, and we have decided to include them in the manuscript. At the beginning of the method section, we now introduce the model as follows:

Lines 91-101: ‘To understand the impact of contract tracing on the epidemic we model

transmission rates as a product of the average number of contacts, where quarantine is considered in a compartmental model similarly to what done by Lipsitch et al, and later followed by several others (e.g., Safi et al 2010, Barua et al 2021, Mubayi et al 2010). One of the factors that makes COVID-19 particularly difficult to trace is the abundance of asymptomatic individuals in the population, who are spreading the disease without awareness of their infectious status (He et al, 2021). Thus, in our model we differentiate infectious cases into three subclasses, namely pre-symptomatic, symptomatic, and asymptomatic (analogously to Miller et al, 2020). While in Lipsitch et al 2003 individuals are quarantined based on contact with infected individuals, we assume that individuals are quarantined based on contact with symptomatic individuals only. Indeed, individuals developing symptoms are more likely to seek testing and become aware of their infectious status.'

2. Some of the parameter values in Table 1 could be supported by available data.

We now explain that some parameters are based on restrictions and demographic of the province of Newfoundland and Labrador (Canada), as follows:

Lines 116-120: 'Model parameters are explained in Table 1. Parameters are based on the population size and public health regulations of the Canadian province of Newfoundland and Labrador, as this province lends itself to this study for having followed an elimination approach to reduce COVID-19 transmission, and having implemented extensive contact tracing and quarantine measures to end transmission whenever an outbreak has occurred.'

We have also added a note in the table reporting the parameter values, to clarify their origin.

We have provided a reference for the range of probabilities of infection given a contact and for the range of contact rates considered (parameters alpha and c).

A couple of minor issues:

3. To follow the typical structure of papers in Plos One, I suggest the authors to put the model and calculations in an appendix or supplementary material.

As suggested, model and calculations have now been moved to the appendix, and in the 'model and methods' section we provide now only a brief description of the main features of the model.

The appendix is structured as follows:

Appendix A) Model description

A.1: The underlying SEAIR model

A.2 Modelling contact tracing

A.3 Modelling immunity

A. 4 Modelling delays in outbreak detection

A.5 Modelling case importations

A.6 Quarantine effectiveness

Appendix B) Contact tracing efficiency and controllable outbreaks

B.1) Minimal contact tracing efficiency

B.2) Contact tracing capacity

4. Formula (11) for the cumulative number of quarantined is not clear to me, please clarify.

We clarified the derivation of this formula in Appendix A.6. The formula provided in the previous version of the manuscript was missing an integral, as pointed out by reviewer 2, and we think that this issue may be the reason why the formula was unclear. We hope that the formula and its explanation is clearer now.

5. The manuscript may be checked to correct some minor issues. Some examples:

- "an analytical criteria" in the Abstract

- p.9, l.-5: When -> when

The manuscript has been rechecked for grammar errors and typos.

Reviewer #2: The paper "Downsizing of contact tracing during COVID-19 vaccine roll-out" investigates the interplay between vaccination and contact tracing in controlling the still ongoing COVID-19 pandemic.

The authors model the epidemic dynamics with a system of delay-differential equations which extends a previously published model by quarantine orders due to contact tracing and the effects of vaccination. Within this model the authors, by solving the system of delayed differential equations numerically, explore in several scenarios what measures are necessary to avoid overwhelming the capacities of contact tracing (and thus a major outbreak).

The main messages of the paper are then that for contact tracing to be effective it has to be quick and efficient, vaccination supports contact tracing by reducing the efficiency of tracing necessary to keep the epidemic under control and that imported cases in populations with low vaccination quotas may quickly overwhelm contact tracing capacities.

The paper is well written and its methods and derivations are easy to understand and follow. The findings of the paper are largely supported by the evidence that the authors present. I recommend that the paper is accepted after the authors address the following points.

I have attached a .pdf file that contains my major and minor remarks.

Major Remarks

1. The title of the paper and the discussion suggest that the downsizing of contact tracing occurs simultaneously to the roll-out in of the vaccine and lifting of contact restrictions. Contrasting this, the paper deals with the situation where a fixed amount of the population is already vaccinated, contacts remain constant and contact tracing is only implemented after the fact. This should be formulated more explicitly in the title as well as introduction and discussion section of the paper.

We agree with the reviewer that contact tracing is only implemented after a certain proportion of the population is already vaccinated, and contact tracing downsizing does not occur simultaneously to vaccine roll-out. Additionally, we think that referring to 'immunity' (acquired either through natural infection or through vaccination) rather than to 'vaccination' may be a better choice, especially given the low level of immunity conferred by vaccination against different viral variants. To this regard, we changed the title as follows:

‘Downsizing of COVID-19 contact tracing in highly immune populations’.

And substituted ‘vaccine roll-out’ with ‘immunity’ or ‘vaccination’ when necessary throughout the manuscript.

2. The conclusion after Equation (17), namely that $q*$ is always smaller than 1 is mistaken. Indeed, take pc very small, then the right-hand-side of Equation (17) blows up. Additionally for $q*$ to be known analytically one would have to calculate the time-varying probabilities pIc , pIp , pIa which, to my knowledge, one cannot. The authors should thus formulate this more carefully in the discussion section of the paper, especially the sentence ”Analogously, we show that, when highly efficient, contact tracing alone can be considered an effective control measure even in the absence of vaccination or social distancing”.

We thank the reviewer for pointing out this problem with our approximation. We now specify that contact tracing alone can be considered an effective control measure only when q* is between 0 and 1, which is the case for the parameter space considered. For example, we write:

Lines 141-145 : ‘For 0 < q^*_0 < 1 there exists a minimal contact tracing efficiency that can prevent infection spread, even in the absence of social distancing requirements, and independently from the immunity status of the population. In this case, contact tracing can be considered an effective sole intervention against COVID-19.’

Lines 148-152: ‘We find that, for the parameter space considered, when the contact tracing efficiency q is large enough (i.e., q < 0.5 in Fig 2), overwhelming contact tracing capacity does not occur, independently from the contact rate and immunity status of the population, and contact tracing alone is a sufficient measure to keep epidemic spread under control.’

Additionally, the derivation of Eq. 1 has now been moved to the Appendix, as suggested by reviewer 1.

For clarity, we have also introduced a mathematical terminology to distinguish between the contact tracing efficiency q, the minimal contact tracing efficiency required to avoid a growth in the number of cases q*_0 (determined analytically) and the minimal contact tracing efficiency required to avoid overwhelming contact tracing capacity q*_c (determined numerically in Fig. 2).

3. While some of the parameters in Table 1 stem from references, others are based on estimates or choice of the authors, e.g. the total population size N, the probability of infection given a contact α, the contact rate c and the amount of imported cases m. Indeed, a back-of-the-envelope calculation (setting q to 0) yields

$$R0 \approx c\, r\ \ bp\, \delta Ip + bc\, \delta Ic\ + c\,(1 - r)\, ba\, \delta Ia = 5.5$$

which seems more in line with the Delta variant than the original strain. The text should elaborate more on the reasons for these specific values, ideally citing studies (especially for the critical parameters α and c).

Reviewer #1 raised the same issue, and we provided a complete response above (see comment 2). We have now indicated that some parameters are based on restrictions and demographic of the province of Newfoundland and Labrador (Canada), and we have provided additional references for parameters alpha and c.

4. The contact tracing efficiency q is never formally introduced and differs in meaning across the paper; in Equation (5) it is meant as the fraction of symptomatic cases whose contacts are traced while in Section 4 it is interpreted as the fraction of contacts of infected cases that are traced. While this distinction does not matter mathematically it should either be clarified what the interpretation of q is or argued that one can interpret it both ways.

We have now defined the contact tracing efficiency at lines123-124:

'We define the contact tracing efficiency q as the proportion of symptomatic individuals whose contacts will be traced multiplied by the proportion of contacts that will be quarantined.'

And again in the appendix, at lines 453-457.

5. If Equation (11) is to be the "cumulative size of Sq", then it is missing an integral (from 0 to T). Indeed setting S0 = N we see that Equation (11) is the same as the first term in Equation (9g). The same reasoning applies to the Section on quarantine effectiveness in the Appendix (e.g. the right-hand sides of Equations (18) and (19) still depend on t). The authors should check how this affects the simplification presented in Equation (19) (one cannot cancel Et−5 anymore) and the results presented in Figure 4.

We thank the reviewer for pointing out this issue. We have now corrected both equations (corresponding to Eqs. (13) and (14) in the revised version of the manuscript). Fortunately, despite the formula having been reported incorrectly in the manuscript, the cumulative sizes of

Sq and Q were computed correctly in the simulations. Therefore, our results remain unaffected.

We agree that the simplification provided in the original manuscript was unclear. We have now clarified our definition of quarantine effectiveness, presented in Appendix A.6.

Minor Remarks

1. In Section 3.1 the parameter pIc is sometimes called pc, this should be unified.

Thank you, we have now unified as p_{I_c} for all entries.

2. Before Equation (17) one actually requires α c S0 N to be big (relative to ˜δ and pc) to arrive at Equation (17); so higher vaccination coverage and lower probability of infection make the approximation worse.

Thank you, at lines 137-139 we now mention that the approximation requires low proportion ofo immune individuals and a high proportion of symptomatic cases.

3. The right-hand side of the system of equations (9a-9h) is discontinuous, as q depends on Ic and, when considered, imported cases cause discontinuities in the solution. Clarification on how the authors dealt with these discontinuities would allow the presented results to be more reproducible.

Thank you for this suggestion. To deal with the discontinuity, we modeled the Heaviside function as a continuous inverse tangent function. We explain now this at lines 500-504. Additionally, in Appendix A we now provide a sketch of the inverse tangent function used (Fig. A.1). To ensure reproducibility, the computer code used is now publicly available in Figshare.

Lines 500-504:

'Because the system of Eq. 10 presents a discontinuity in parameter q (see Eq. 4), for the simulations we choose to substitute the Heaviside function with the continuous inverse tangent function shown in Fig. A1. The computer code is publicly available at: https://figshare.com/articles/figure/Code_for_Figures_of_the_manuscript/19103183.

4. The reference Miller et al. 2020 has already been published (albeit under a slightly different name), the authors should cite the published version.
Thank you, we now cite the published version which had, indeed, a slightly different title.

5. Some references, e.g. Davies et al. 2020, Davis et al 2021, Ferretti et al 2020, use ”covid-19” and ”sars-cov-2” instead of ”COVID-19” and ”SARSCoV-2”, the authors should fix the spelling in the references.

Thank you, we have corrected these entries in our references.

6. The beginning of Section 2.2 reads ”As only symptomatic individuals are contact traced ...”. With the more widespread availability of rapid tests pre- and asymptomatic cases can be identified early on which would allow to have their contacts traced as well. I suggest that the authors add a rationale on why only symptomatic individuals are contact traced.

We have added the following sentence (lines 101-103)

‘We consider asymptomatic and pre-symptomatic individuals unlikely to seek testing, especially as we consider the situation where the community is not experiencing any outbreak prior to disease introduction.’

Additionally, at the end of the discussion we mention the following (lines 274-278):

‘Contact tracing could become more efficient by considering that pre-symptomatic and asymptomatic individuals, once identified as positive contacts of a symptomatic case, can as well contact trace. This particular feature could add realism to the model, but further complicate its formulation, and it will therefore be left for future work.’
